# Squamous Cell Carcinoma Antigen: Clinical Application and Research Status

**DOI:** 10.3390/diagnostics12051065

**Published:** 2022-04-24

**Authors:** Huange Zhu

**Affiliations:** Department of Clinical Laboratory, The Second Affiliated Hospital of Xi’an Jiaotong University (Xibei Hospital), Xi’an 710004, China; zhg1990@mail.xjtu.edu.cn

**Keywords:** squamous cell carcinoma antigen, tumor marker, serine protease inhibitors, diagnosis, prognosis

## Abstract

The squamous cell carcinoma antigen (SCCA) is a tumor marker that has gained increasing attention for its biological functions and significance in normal physiological and pathological processes. Not only SCCA but also circulating immune complexes of SCCA and immunoglobulin M (IgM) are involved in normal physiological and pathological processes, providing a background for numerous clinical studies aimed at assessing the potential role of SCCA, SCCA–IgM, and SCCA isoform complexes in clinical practice. Previous studies support the clinical value of SCCA as a tumor marker for either diagnosing squamous cancers or monitoring the response to radiotherapy or chemotherapy, tumor relapse, and treatment failure. However, these studies show contrasting results, making the diagnostic or prognostic value of SCCA controversial. To reduce clinical heterogeneity across studies and achieve a more accurate and reliable comparison of results, a standardized detection method, scoring system, and cutoff level need to be established. Moreover, despite the fact that performances of different methods are comparable, the dynamic observation of tumor marker kinetics should be conducted under the same method.

## 1. Introduction

The squamous cell carcinoma antigen (SCCA), a tumor-specific antigen, was first isolated by Kato and Torigoe from squamous cell carcinoma (SCC) tissues of the uterine cervix in the 1970s [1]. SCCA consists of two highly homologous isoforms, SCCA1 and SCCA2, encoded by *SERPINB3* and *SERPINB4* genes, respectively, and is located on the long arm of chromosome 18 (18q21.3). SCCA1 and SCCA2, also termed SERPINB3 and SERPINB4, respectively, belong to the serine protease inhibitor family (SERPINBs) and consist of an ovalbumin-like domain with nine α-helices and three antiparallel β-sheets, and a reactive center loop that is essential for binding and inhibiting the target protease [2].

**Figure 1:**
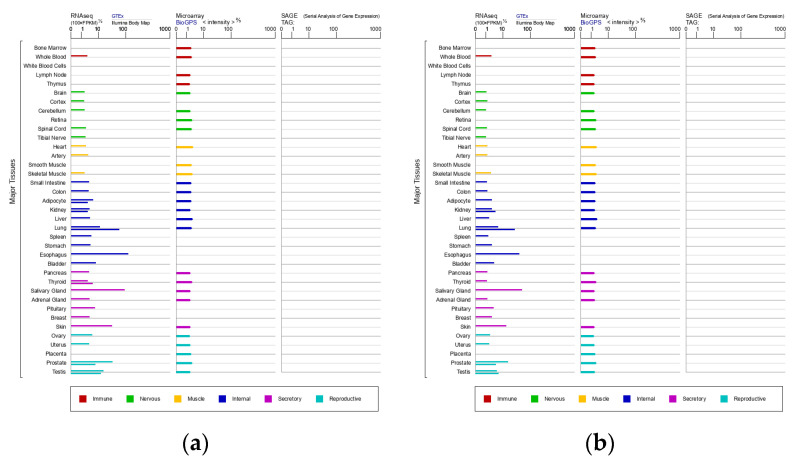
Expression of SCCA1 (**a**) and SCCA2 (**b**) in major tissues.

SCCA1 and SCCA2 share a 98% degree of homology and are 92% identical at the amino acid level. SCCA1 is the neutral form of SCCA (pI = 6.4), while SCCA2 is the acidic form (pI = 5.9) [3]. Normal squamous epithelium cells can express SCCA, and SCCA1 and SCCA2 are usually co-expressed in the same tissues of the immune, nervous, muscular, secretory, and reproductive systems and internal organs, as shown in Figure 1 from GeneCards, a database of human genes (www.genecards.org, 13 January 2022) [4]. A genetic variant of SCCA has been reported in a minority of hepatocellular carcinoma (HCC) cases [5,6]. SCCA1 mainly inhibits papain-like cysteine proteases, while SCCA2 inhibits chymo-trypsin-like serine proteases [7]. These isoforms may be differently expressed in tumors and skin diseases [8].

**Figure 2:**
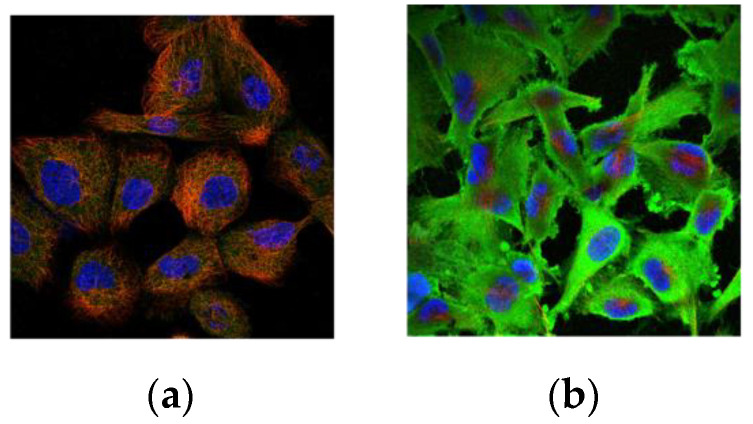
Subcellular localization of SCCA1 in human cells: (**a**) cytosol (CAB036006:A-431); (**b**) membrane and cytosol (CAB036006:U-251 MG).

SCCA1 and SCCA2 are mainly located in the cytoplasm. However, this precise subcellular localization may change under various conditions, as these isoforms can also be detected in the cytosol, nucleus, plasma membrane, or lysosomes or extracellularly, which suggests their precise biological functions [9,10,11]. Figure 2 shows typical images of the subcellular localization of SCCA1 in human cells from the Human Protein Atlas (www.proteinatlas.org, 13 January 2022).

Factors affecting the detectability of SCCA in serum have been determined by researchers. These include:

- Tumor size and volume (more tumor cells result in a larger amount of SCCA);
- Invasiveness of the primary tumor or recurrence;
- Lymph node metastasis (LNM) (secreted SCCA from tumor cells in lymph nodes is easily detected in the bloodstream [12]);
- Distant metastasis (circulating tumor cells enable easy detection of secreted SCCA in blood tests [13]);
- Impairment of immunosurveillance.

The expression of SCCA in peripheral T-lymphocytes indicates that both tumor cells and T-lymphocytes may cause the production of serum SCCA [14]. Additionally, the existence of immunoglobulin M (IgM) contributes to the formation of SCCA–IgM complexes.

Even though SCCA testing is routinely performed to help clinicians diagnose and manage diseases, studies have shown conflicting results regarding its usefulness. Our work aims to review studies to review studies of SCCA in recent years, discuss the potential causes of consistent and inconsistent results, and explore the possible solutions.

## 2. SCCA Measurement

The demand for routine clinical SCCA testing has rapidly increased because of its vital role in the diagnosis and prognosis of SCCs and other diseases. Detection methods have also improved with the development of biotechnology. The low degree of biochemical diversity of biotechnological products has enabled the generation of monoclonal antibodies specific for either SCCA1 or SCCA2, allowing separate measurements of each instead of total SCCA. Common methods used to detect SCCA levels in serum and tissue include radioimmunoassay, enzyme-linked immunosorbent assay (ELISA), Western blot (WB), immunohistochemistry (IHC), and immunoluminometric assay.

One of the first methods to detect SCCA was radioimmunoassay [1,15]. Although radioimmunoassay is sensitive and reliable, it has numerous disadvantages including radiation exposure and poor reagent stability, gradually making its use less widespread.

ELISA, a test based on antigen–antibody reaction and color change to identify protein levels, used to be the most common method for detecting SCCA. However, ELISA also came with disadvantages, including poor repeatability, a narrow linear range, poor detection efficiency, and tedious experimental procedures; thus, it is not sufficient to meet the clinical demand.

The basic principle of WB consists of using specific antibodies to target proteins in cells or biological tissues during gel electrophoresis and analyzing the molecular size of the proteins in relation to the reference ladder to measure protein expression. The complex experimental procedures and high requirements for WB have also limited its use for large-batch testing in the clinic. However, because of its high specificity and accuracy, WB remains a method of choice in research studies. Meanwhile, IHC is a method applying the principles of immunology and histochemistry to qualitatively or quantitatively identify components in cells or tissues in situ. Both WB and IHC are only suitable for small samples.

Recently, automated non-radioimmunoassay methods have been performed to measure SCCA levels. Electrochemiluminescence immunoassay (ECLIA) with the Roche Cobas e602 system and chemiluminescent microparticle immunoassay (CLIA) with the Abbott ARCHITECT i2000 system are two common automated systems used in the clinical laboratory. Although there is a strong positive correlation (r = 0.9658) between these two systems, the tested SCCA levels are reportedly higher with the Roche system than with the Abbott system [8]. These differences might be attributed to the different serum proteins and heterophilic antibodies used [16] and different reactivities against antigens among specific antibodies produced by various manufacturers [17]. Reportedly, the Roche system can detect SCCA1 and SCCA2 in an equimolar manner [18], while the Abbott system cannot determine the proportions of SCCA1 and SCCA2 [8] or can only detect SCCA1 but not SCCA2 [19]. Manual ELISA and automated ECLIA have shown comparable performances in SCCA detection [20]. However, despite comparable performances between different methods, the dynamic observation of tumor marker kinetics should be performed under the same method.

## 3. SCCA Levels in Cancer and Inflammation

The biological functions and significance of SCCA in normal physiological and pathological processes have been widely studied. Besides SCCA, circulating immune complexes of SCCA and IgM have also been reported in normal physiological and pathological processes, providing a background for studies investigating the potential role of SCCA, SCCA isoforms, and SCCA–IgM complexes in clinical practice [21] (Table 1).

A recent study on the relationship between SCCA and 39 different clinically defined diseases shows that SCCA is a clinically used biomarker not only for SCC but also for other human diseases [7].

### 3.1. Cervical Cancer

Imaging techniques, including computed tomography (CT) and positron emission tomography (PET)/CT, have been increasingly applied in the clinical diagnosis and treatment of cervical cancer; however, such methods have limitations, including false-negative and false-positive results [22,23]. Nevertheless, the diagnostic efficacy of combined PET/CT with serum SCCA in the diagnosis of early recurrent cervical cancer is higher than that of either PET/CT or serum SCCA methods alone [24,25]. Thus, this combined method is clinically valuable in the diagnosis of early postoperative metastases and recurrence in cervical cancer.

Ryu HK et al. [26] reported that SCCA levels > 1.86 and >0.9 ng/mL were the optimal pretreatment and posttreatment cutoff values for predicting recurrence, respectively, and were significantly associated with poor disease-free survival (DFS). With a high risk of cancer recurrence, closer surveillance is thus needed after complete remission therapy [26]. Another study reported 4, 1.5, and 4 ng/mL as the best cutoff values for SCCA at pretreatment, treatment, and recurrence, and that the predicting value of SCCA after treatment and at recurrence for recurrence and survival is significant only when pretreatment SCCA levels ≥ 4 ng/mL [27].

Multivariate analysis, particularly of surgical treatment for early-stage cervical SCC (stages IB–IIA), demonstrated that SCCA levels ≥ 2.75 ng/mL can be used as a potential marker to predict LNM in early stage cervical cancer preoperatively [28]. Another study investigating the utility and cutoff level of serum SCCA to predict LNM in locally advanced cervical cancer showed that the SCCA level significantly correlated with paraaortic lymph node status, but not with pelvic lymph node status and parametrial involvement [29]. In another study, the positive lymph node rate of patients with pretreatment SCCA levels ≥ 3.9 ng/mL significantly decreased after neoadjuvant chemotherapy (NACT). The overall survival (OS) was significantly longer in the NACT group than in the conventional group (with radical surgery alone) when the pretreatment SCCA levels were ≥4.55 ng/mL [30].

Not only are elevated pretreatment SCCA levels associated with radiotherapy resistance, extensive tumors, and poor survival in cervical cancer patients treated with definitive concurrent chemoradiotherapy (CCRT) but also the rate of SCCA reduction during CCRT can predict tumor response after treatment [31]. SCCA also shows high sensitivity for the early detection of cervical cancer relapse during follow-up after CCRT, and it is cost-effective [31]. Considering the optimal posttreatment SCCA level cutoff of 1.8 ng/mL for predicting treatment failure, patients with posttreatment SCCA levels ≥ 1.8 ng/mL are more likely to come to treatment failure and poor survival [32].

Even with the potential benefit of monitoring SCCA during follow-up of early cervical cancer patients after treatment, the cure rate of patients with elevated SCCA levels does not seem to improve, partly owing to the lack of curative salvage treatments [33].

A novel model of measuring kinetic change in SCCA levels before surgery, and on days, weeks and months after surgery [34], and their associations with clinicopathologic characteristics show that SCCA levels decreased dramatically after surgery and postoperative SCCA could keep stable or fluctuate to some extent within a half-year [34]. The SCCA levels preoperatively and at earlier time points postoperatively were higher in patients with risk factors. The kinetic trend of SCCA might be mainly affected by postoperative adjuvant therapy, as the SCCA levels reached the same level between the low-risk, intermediate-risk, and high-risk group after completion of treatment [34]. A new model of SCCA, such as SCCRR (pre-RT SCCA − mid-RT SCCA/pre-RT SCCA × 100) [35] was identified as an independent and strong prognostic parameter for patients with cervical cancer receiving radiation therapy (RT). Chang et al. [36] established that the hazard at time t is dependent on SCCA value at the same time and that patients should be followed with routine SCCA assessments, and the time-dependent SCCA is the best predictor of both relapse and death.

The failure of posttreatment SCCA levels to normalize may predict tumor relapse, and adjuvant therapies should be considered for these patients [31]. There is a much higher percentage of early stage (FIGO stage IB/IIA) cervical cancer patients with elevated preoperative serum SCCA (>1.9 ng/mL) who had postoperative indications for adjuvant radiotherapy than of patients with normal preoperative SCCA. For IBI patients with no indications for adjuvant radiotherapy, the percentage of patients with elevated preoperative serum SCCA was much higher than that of patients with normal serum SCCA levels relapse within 2 years [37].

On the one hand, surgery is the standard treatment for early-stage cervical cancer patients. On the other hand, postoperative concurrent chemoradiotherapy is recommended for patients with any high-risk features, such as LNM, parametrial involvement, and positive surgical margins. However, for tumor patients with a combination of intermediate-risk factors such as large size, lymphovascular involvement, and deep stromal invasion, there is no consensus on whether adjuvant chemotherapy should be administered to those with two intermediate-risk factors, as this may lead to over or under treatment. A study indicated that preoperative SCCA can be a predictive marker for the use of adjuvant chemotherapy in cervical SCC with intermediate-risk factors [38]. It indicated that for patients with a high pretreatment SCCA level, those who received adjuvant chemotherapy have a better prognosis and a lower rate of distant metastasis than do patients who did not receive chemotherapy; this is not observed in patients with a low pretreatment SCCA level [38].

Another study found that cervical cancer patients with elevated pretreatment SCCA did not benefit from adjuvant chemotherapy and a considerable proportion of these patients had postoperative indications for adjuvant radiotherapy [39]. For these cervical cancer patients with elevated pretreatment SCCA, the choice of radical hysterectomy and adjuvant chemotherapy should be prudent [39].

During follow-up of cervical cancer patients treated with radiotherapy, renal dysfunction was significantly associated with a greater incidence of SCCA elevation [40].

### 3.2. Lung Cancer

SCCA has also been widely used as one of the tumor markers for monitoring non-small cell lung cancer (NSCLC), although recent reports challenge its value in routine test because of low sensitivity [41].

As the incidence of peripheral SCCs of the lung (p-SqCCs) has increased over recent years, multivariate analysis of p-SqCCs patients revealed that serum SCCA is also an independent prognostic factor for completely resected p-SCCs; stage T1 p-SCC with a high serum SCCA level or vascular invasion should be upgraded to T2a, which accurately reflects survival status of patients with p-SqCCs [42].

SCCA1 was identified as a predictive biomarker for response to platinum combination chemotherapy (PtC) and also as an independent prognostic value for untreated patients with resected NSCLC. SCCA1 expression strongly correlates with clinical response in PtC-treated NSCLC patients [11]. On the one hand, a targeted therapy is not currently available for most patients with advanced-stage disease and PtC remains a key part of the systemic treatment for them. On the other hand, a SCCA1 score of ≥2 [43] identifies a subgroup of patients with stage IV NSCLC who have a poor survival when treated with PtC, similar to that estimated of untreated or chemorefractory stage IV NSCLC, indicating SCCA1 as a useful biomarker that identifies a highly resistant subgroup for whom PtC should be avoided [43].

SCCA has not only been found in patients with SCC but also in patients with non-squamous cell lung cancer (NSCC) and nonmalignant pulmonary disease (NMPD). However, SCCA may serve a contradictory role in different types of NSCLC. SCCA1 is significantly decreased in metastatic lesions when compared with that in paired primary SCC, supporting an inhibition role of SCCA1 in tumor invasion and metastasis; this phenomenon does not exist in AC. High SCCA1 expression indicates poor prognosis in AC, and SCCA1 may potentially function primarily as a negative regulator of cell death in AC. The contrasting value of SCCA1 in AC and SCC, indicates a dual pathogenic role of SCCA1 in different histologic types of NSCLC [11]. Although SCCA is still a widely used tumor marker for monitoring NSCLC, a study reevaluated the efficacy of SCCA and CYFRA21-1 in diagnosing NSCLC. The sensitivity of SCCA is significantly lower than that of CYFRA21-1 for patients with both NSCLC and metastasis, and the combination of SCCA with CYFRA21-1 can only induce a minimal increase in CYFRA21-1 sensitivity [41]. This indicates that SCCA should be considered as an inefficient factor for NSCLC diagnosis.

Elevated SCCA levels (>1.5 ng/mL) have been reported in SCC (52.7%), NSCC (14.2%), and NMPD (28.4%) patients. However, serum SCC levels of ≥40.0 ng/mL in NSCC patients and ≥20.0 ng/mL in NMPD patients have not been observed [44]. Interstitial lung disease (ILD) is the major determinant of prognosis of patients with systemic sclerosis (SSc). With a cutoff value for serum SCCA–IgM at >200 AU/mL, SCCA–IgM was significantly higher in SSc patients with ILD, which is a major determinant of the prognosis in SSc patients, than in those without ILD [45].

### 3.3. Neck and Head Cancer

Even though studies have recently been conducted to identify the correlation between SCCA and clinicopathologic features in head and neck SCC (HNSCC) patients to evaluate the clinical usefulness of serum SCCA in the management of patients with HNSCC, the role of SCCA in these patients remains controversial.

The pretreatment serum SCCA2, not SCCA1, was significantly higher in HNSCC patients than in controls [46]. Another study indicated that SCCA1 is highly active in oral SCC but nearly undetectable or expressed at a low level in normal tissues [47].

SCCA levels have a significant correlation with male sex and TNM stage, and SCCA is significantly higher in patients with advanced T and N classification tumors. One analysis indicated that it may not be used as a predictive marker for OS and DFS in HNSCC patients [48], while another revealed that SCCA was a significant risk factor against OS in cancers of the oral cavity, hypopharynx, and larynx, but not in oropharyngeal cancer [13]. SCCA was higher in lymph nodes of HNSCC patients than in those of other patients, and fine needle aspiration of cervical lymph node samples containing SCCA is a reliable test for detecting HNSCC and precedes an even more accurate detection of HNSCC LNM when used in addition to fine needle aspiration cytology [49].

Inverted papilloma (IP) is a benign neoplasm, although it has a high tendency to recur or develop malignancy. It is vital to identify it from inflammatory diseases. Elevated serum SCCA levels were observed in 83.3% of patients with IP regardless of whether this being new or recurrent and in 5.3% of inflammatory patients, and the sensitivity and specificity of diagnosis IP from IP and inflammatory groups, based on SCCA levels (1.5 ng/mL), were 83.3% and 94.7%, respectively [50]. Another study indicated that the sensitivity and specificity were 80.0% and 93.3% for IP diagnosis, respectively, based on the SCCA level (>1.5 ng/mL) in IP and nasal polyp groups [51]. Although serum SCCA levels were elevated in both IP and sinonasal SCC groups, the distribution and proportion of SCCA1 and SCCA2 were distinct in these patients. Patients with sinonasal IP predominantly express SCCA1, whereas those with sinonasal SCC predominantly express SCCA2 [52].

Yamashita et al. [50] also found that smoking and tumor volume significantly correlated with SCCA levels in IP. Several studies have shown that postoperative rather than preoperative SCCA levels significantly decreased in the IP group [50,51,53]. Even though postoperative SCCA levels were positively associated with future recurrence, with a good ability to identify recurrence, no correlation between SCCA levels and recurrence during follow-up was found [53]. The high postoperative SCCA level might be induced by residual disease, and most recurrence events develop at the site of the original tumor, indicating that residual disease is the main cause of recurrence [53].

SCCA1 is not only associated with prognosis and chemoresistance but also with the microenvironment in esophageal adenocarcinoma (EAC) [54]. SCCA1 and SCCA1–IgM serum levels were significantly higher and lower, respectively, in EAC patients than in healthy controls. Even though SCCA–IgM and free SCCA1 were inversely correlated with immune activation markers, only free SCCA1 was significantly associated with worse OS. In vitro, EAC cell lines overexpressing SCCA1 showed significantly more resistance to cell death induced by different chemotherapeutic drugs [54], and human recombinant SCCA1 was shown to induce a significant increase in the inhibitory molecule PD-L1 levels in monocytes in vitro. Aside from tumor cells, SCCA was also expressed in peripheral T-lymphocytes, indicating that both tumor cells and T-lymphocytes may contribute to increased serum SCCA [14]. Therefore, the reduced tumor chemosensitivity and intra-tumoral immunity impairment induced by SCCA might a contributing factor to poor prognosis in EACs overexpressing SCCA1 [54].

### 3.4. Liver Cancer

SCCA has also been found to be elevated in liver cancer and has been detected as SCCA–IgM complexes in serum of hepatocellular carcinoma (HCC) patients [21].

Reliable evidence shows that SCCA expression progressively increases during hepatocarcinogenesis, i.e., from chronic liver disease to dysplastic nodules to HCC [55]. Tumoral SCCA, but not peritumoral or serum SCCA, is dependent on nodule size, and there was an inverse correlation between nodule sizes and SCCA levels [56]. The significantly stronger expression of SCCA in smaller than in larger HCC could be important for early HCC detection [56,57]. While a positive correlation was observed between tissue and serum levels of SCCA in liver cirrhosis patients, there was no such correlation in HCC [56]. Interestingly, Li et al. [6] reported the rate of SCCA2 expression was much higher than SCCA1. Altogether, SCCA only shows moderate diagnostic accuracy for HCC screening, possibly because SCCA is also increased in liver cirrhosis and chronic liver disease [58].

An increasing number of studies have shown that both pretreatment serum AFP and SCCA–IgM levels were significantly higher in HCC patients than in patients with cirrhosis [59]. The combination of SCCA–IgM complexes and AFP has greater sensitivity than either biomarker alone, particularly for patients with AFP values between 20–200 ng/mL [57]. The serum SCCA–IgM level was significantly lower in HCC patients who underwent surgical resection than in those who received other therapy (TACE, RFA, or palliative care) [59]. This decrease in SCCA–IgM levels might be caused by the removal of cancer tissues or the reduction in liver damage and associated immune response [59]. Serum SCCA–IgM levels at baseline and 1 month after treatment were significantly lower in patients who responded to therapy than in those who did not respond [60], suggesting that SCCA–IgM level determination could help predict the response to therapy in HCC patients.

Circulating SCCA–IgM was more frequently detected in patients with chronic hepatitis C (CHC) than in those with negative hepatitis C virus (HCV) [61]. In HCV-positive patients, there is a significant correlation between the SCCA1 level in the liver and the serum SCCA–IgM level. The kinetics of SCCA–IgM is related to antiviral therapy. In patients with sustained virologic response, the SCCA–IgM level decreased significantly after treatment for a half-year and remained persistently low even a half-year after the last therapy [61]. In contrast, although non-responders showed a significant decrease after a half-year of treatment, SCCA–IgM reached almost initial values after a half-year of therapy withdrawal. As previous studies have shown that nonalcoholic steatohepatitis (NASH) occurs frequently in HCV infection and is recognized as an increasing risk factor for liver disease and HCC development, SCCA–IgM may be useful for the identification of HCV-infected patients with a high risk of disease progression and HCC development [61,62]; SCCA–IgM and HCV genotype 3 have a significant association with the presence of NASH. In contrast, SCCA–IgM does not seem to have a role in the identification and prognosis of nonalcoholic fatty liver disease in patients with obesity, prediabetes, and diabetes undergoing sleeve gastrectomy [63].

SCCA–IgM baseline of patients who developed HCC were significantly higher than patients who did not during the same follow-up period, especially in HCV-infected patients, and positivity of SCCA–IgM at baseline was associated with a significantly shorter HCC-free survival [64].

SCCA–IgM is a dynamic biomarker, alternating as the disease progresses [21] and over therapy process [60]. Accordingly, monitoring of SCCA–IgM variations before and after therapy or at different time points might be more useful than monitoring at a single time point, and it can provide a better understanding of disease progression [21,60].

### 3.5. Inflammation

Recent studies have indicated the usefulness of monitoring SCCA levels in pediatric and adult atopic dermatitis (AD). Serum SCCA levels in the psoriasis group was significantly higher than those in controls and significantly decreased after treatment [65]. Both SCCA1 and SCCA2 were higher in AD patients than in volunteers and positively correlated with the clinical severity of AD [19,65]. However, with the predominant expression of SCCA1 and SCCA2 in cervical cancer and AD patients, respectively, detection and discrimination between SCCA1 and SCCA2 are critical in estimating the severity of AD and distinguishing AD from other cancers [19].

Serum SCCA2 levels were significantly higher in patients with lichen planus (LP) than in healthy controls, as well as in female patients than in male patients [66]. The mean serum SCCA2 levels were significantly higher in patients with eruptive LP than in those with localized and hypertrophic forms. Further studies are needed to assess the therapeutic effect of SCCA2 blockade, which could improve outcomes of LP patients [66].

Watanabe et al. [67] have stated that even though SCCA1 and SCCA2 highly correlated, SCCA2 may have much better clinical usage. Consistent with previous studies, their study showed that serum SCCA2 levels were significantly higher in psoriasis patients than in healthy controls and correlated well with disease severity and reflected treatment efficacy. Only serum SCCA2 showed a significant increase in AD when assessed in each age group or in subgroup analysis [67,68]. Increased SCCA2 staining was observed in the lesional skin of psoriasis patients, and skin SCCA2 levels correlated with serum SCCA2 levels. The underlying mechanism for the significant relation between SCCA2 level and disease severity remains unclear yet. An earlier study showed that keratinocytes produced mainly SCCA2 with stimulation of IL-4 or IL-13, IL-17, and IL-22 [19,67].

**Table 1:** Summary of investigation of SCCA in studies.

Ref.	Marker	Disease	Sample Type	Sample Collection Time	Method	Cut-Off	Conclusion
Pontisso et al., 2004 [5]	SCCA1 variant	HCC	Tissues	At surgery	IHC	-	Diagnose HCC
Li et al., 2014 [6]	SCCA1/2, SCCA1 variant	HCC	Tissues	At surgery	RT-PCR Sequencing	-	Diagnose HCC
Lin et al., 2011 [12]	SCCA	OSCC	Serum	Preoperative	CLIA	2.0 ng/mL	Predict metastasis, DFS and OS
Imai et al., 2015 [13]	SCCA	HNSCC	Serum	Pretreatment	CLIA	1.1 ng/mL	Predict survival
Beneduce et al., 2005 [21]	SCCA-IgM	HCC	Serum	Pretreatment	EIA/WB	120 AU/mL	Diagnose HCC
Ryu et al., 2015 [26]	SCCA	CSCC	Serum	Pretreatment Posttreatment	-	1.86 ng/mL 0.9 ng/mL	Predict recurrence
Choi et al., 2020 [27]	SCCA	CSCC (stage IB-IVA	Serum	Pretreatment Treatment Recurrence	IRA	4 ng/mL 1.5 ng/mL 4 ng/mL	Predict recurrence and survival
Zhu et al., 2021 [28]	SCCA	Early CSCC	Serum	Preoperative	CLIA	1.5 ng/mL	Predict LNM and survival
Lekskul et al., 2015 [29]	SCCA	CSCC (stage IB2-IVA	Serum	Pretreatment	CLIA	1.5 ng/mL	Predict pelvic and paraaortic LNM
Chen et al., 2020 [30]	SCCA	CSCC	Serum	Pretreatment posttreatment	ECLIA	3.9 ng/mL 2.7 ng/mL	Evaluate the LNM and prognosis of CSCC who received neoadjuvant chemotherapy
Wang et al., 2019 [32]	SCCA	CSCC	Serum	Posttreatment	CLIA	1.8 ng/mL	Predict treatment failure and poor survival of CSCC who received concurrent chemoradiotherapy
Salvatici et al., 2016 [33]	SCCA	CSCC (stage I-II)	Serum	Posttreatment	CLIA	1.5 ng/mL	Early diagnosis of recurrence
Ye et al., 2020 [34]	SCCA	CSCC (stage IB1-IIA2)	Serum	day 0 (the day before surgery)/postoperative day 4, weeks 2–4, months 2–4 and months 5–7	Single molecule assay (Simoa) prototype immunoassay	2.49/0.66, 0.61, 0.72, and 0.71 ng/mL	Predict disease aggressiveness and treatment response
Chang et al., 2020 [36]	SCCA	CSCC	Serum	Pretreatment Posttreatment	-	-	Predict relapse and death
Reesink-Peters et al., 2005 [37]	SCCA	Early CSCC	Serum		CLIA	1.9 ng/mL	Predict tumor relapse, and guide adjuvant therapies
Guo et al., 2020 [38]	SCCA	CSCC with intermediate-risk factor	Serum	Preoperative	ELISA	6.09 ng/mL	Predict the use of adjuvant chemotherapy
Yuan et al., 2021 [39]	SCCA	CSCC (stage IB-IIA)	Serum	Pretreatment	CLIA	6.09 ng/mL	Guide adjuvant therapies
Oike et al., 2021 [40]	SCCA	CSCC (stage IB-IVA)	Serum	During follow-up	CLIA	1.5 ng/mL	Improve the quality of follow-up and monitor renal dysfunction
Kinoshita et al., 2014 [42]	SCCA	SqCC	Serum	Presurgery	-	1.5 ng/mL	Predict prognosis of resected peripheral-SqCC
Urquhart et al., 2013 [43]	SCCA1	NSCLC (stage IV)	Tissues	Pretherapy	IHC	IHC score ≥ 2	Predict resistance to PtC
Yasumatsu et al., 2019 [46]	SCCA2	HNSCC	Serum	Pretreatment	CLIA	1.5 ng/mL	Predict progression and guide management of HNSCC
Wu et al., 2020 [47]	SCCA1	OSCC	Cell Tissues	-	WB	-	Provide target for OSCC gene therapy.
van Schaik et al., 2019 [49]	SCCA	HNSCC	FNA sample	Pretreatment	CLIA	0.3 μg/mL	Diagnose HNSCC in cervical lymph nodes.
Yamashita et al., 2016 [50]	SCCA	Nasal IP	Serum	Presurgery/postsurgery	CLIA	1.5 ng/mL	Distinguish new and recurrent IP from inflammatory diseases.
Promsopa et al., 2021 [51]	SCCA	IP	Serum	Presurgery/postsurgery	CLIA	1.5 ng/mL	Distinguish IP from patients with nasal polyps and rhinitis.
Yasumatsu et al., 2018 [52]	SCCA1/2	Sinonasal SCC and IP	Serum Tissue	Pretherapy	CLIA	1.5 ng/mL	Distinguish sinonasal IP from squamous cell carcinoma
van Zijl et al., 2017 [53]	SCCA	Sinonasal IP	Serum	Pretreatment/posttreatment	microparticle enhanced immuno assay	2.6/0.8	Predict IP recurrence
Turato et al., 2019 [54]	SCCA1 SCCA-IgM	EAC	Serum Tissue	At surgery	ELISA IHC	156 AU/mL	Predict immune surveillance impairment and reduced chemosensitivity
Trerotoli et al., 2009 [56]	SCCA	HCC	Tissue	Pretherapy	ELISA IHC	-	Early diagnosis of HCC
Bui et al., 2018 [59]	SCCA-IgM	Liver diseases	Serum	Presurgery	ELISA	-	Monitor cirrhosis in an Asian cohort of patients.
Giannelli et al., 2007 [57]	SCCA-IgM	HCC	Serum	Pretherapy Posttherapy	ELISA	-	Increase the accuracy of HCC diagnosis, especially when AFP values in 20–200 ng/mL.
Guarino et al., 2017 [60]	SCCA-IgM	HCC	Serum	at baseline (T0) and one month after treatment (T1)	ELISA	120 AU/mL	Predict the outcome of therapy
Martini et al., 2015 [61]	SCCA-IgM	HCV-infected patients	Serum	Pretherapy Posttherapy	ELISA	-	Identify HCV-infected with a high risk of disease progression and HCC
Biasiolo et al., 2016 [64]	SCCA-IgM	Liver cirrhosis	Serum	During follow-up	ELISA	156 AU/mL	Predict and manage cirrhotic patients at higher risk of HCC development.
Khattab et al., 2020 [66]	SCCA2	Lichen planus	Serum	Pretherapy	ELISA	-	Diagnose lichen planus and predict disease severity
Watanabe et al., 2016 [67]	SCCA2	Psoriasis	Serum Biopsy sample Cellular lysates	serial examinations	ELISA IHC WB	-	Associate with disease severity and reflects treatment efficacy
Takeuchi et al., 2019 [68]	SCCA1/2	Pediatric atopic dermatitis	Serum	Pretherapy	-	-	Diagnose pediatric atopic dermatitis in the Ishigaki cohort.

Abbreviations: CSCC—cervical squamous cell carcinoma; HCC—hepatocellular carcinoma; OSCC—oral-cavity squamous cell carcinoma; HNSCC—head and neck squamous cell carcinoma; SqCC—squamous cell carcinomas (p-SqCCs) of the lung; NSCLC—non-small-cell lung cancer; IP—inverted papilloma; EAC—esophageal adenocarcinoma; IHC—immunohistochemistry; RT-PCR—reverse transcription-polymerase chain reaction; CLIA—chemiluminescent microparticle immunoassay; EIA—enzyme immunoassay; WB—Western blot; IRA—immunoradiometric assay; PtC—platinum doublet chemotherapy; LNM—lymph node metastasis.

### 3.6. Others

Diagnosis of penile cancer, a genitourinary system cancer, is mainly based on self or clinical examination, and confirmatory biopsy [69]. SCCA is the first serum biomarker with clinical use in managing penile cancer [70]. Monitoring SCCA might not only indicate metastases before imaging or clinical examination [70,71], but also be useful in predicting treatment response and DFS of penile cancer patients [72,73]. Serum SCCA can also help to diagnosis bladder cancer, another genitourinary system cancer [8]. Cytoplasmic SCCA, not nuclear SCCA, in bladder cancer cells is associated with bladder carcinoma cells squamous metaplasia [74]. Even a case report of poorly differentiated bladder carcinoma described that SCCA is positive [75]. It has also been reported that *SERPINB4* gene was deleted in all patients of malignant peripheral nerve sheath tumor-like melanoma, a rare malignancy melanoma, and may have a tumor suppressor activity [76].

## 4. Combination of SCCA with Other Markers in Clinical Practice

Apart from the combination of SCCA and AFP in HCC, many studies have also evaluated the value of combining the detection of SCCA and other indicators including CRP, albumin, and noncoding RNAs for clinical use. One study indicated that serum SCCA, hypersensitive C-reactive protein (hs-CRP), and CA125 in the recurrence group of cervical cancer patients were significantly higher than those in the non-recurrence group, with a significant positive correlation between SCCA and hs-CRP, and SCCA and CA125, in the recurrence group patients [77]. Elevated preoperative CRP and SCCA levels adversely influence DFS and OS of oral SCC patients [78]. Preoperative CRP and SCCA levels were also identified as independent biomarkers for LNM, advanced tumor stage, and disease-specific survival in patients with penile SCC [79].

Preoperative serum SCCA and albumin levels can predict survival of esophageal SCC patients with stage T13N0M0, and patients with high SCCA and low albumin levels may have a poor survival outcome [80]. There is a superiority of both miR-215 and SCCA–IgM over AFP in HCC diagnosis, especially in distinguishing HCC with AFP levels < 200 ng/mL and with small-sized focal lesions from cirrhotic patients [81]. In addition, combined pretreatment serum circulating tumor cells (CTC) and pretreatment SCCA demonstrates better predictive accuracy than do the FIGO stage and serum CTC or SCCA alone [82].

## 5. Related Mechanisms

**Figure 3:**
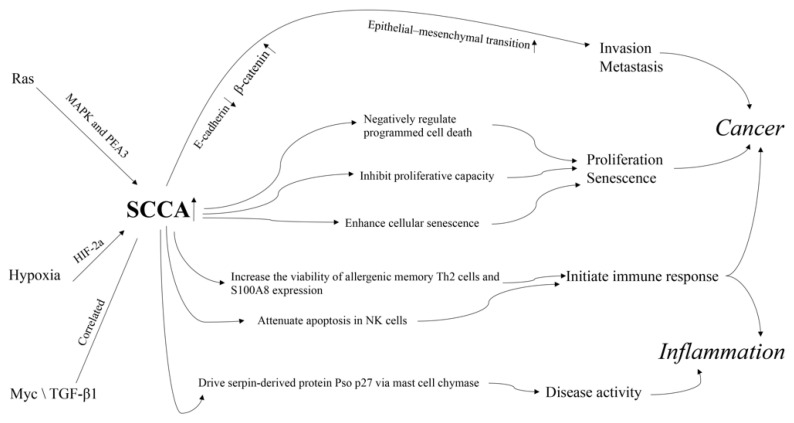
The mechanism of action of SCCA in cancer and inflammation.

Both in vitro and in vivo studies have documented the important roles of SCCA in inflammatory processes and cancer (Figure 3). However, their exact mechanisms remain largely unclear.

First, the abnormal expression of SCCAs is reportedly related to cancer hallmarks and classic tumor related pathways. SERPINB3 can lead to changes in clusters of loosely connected elongated cells, decrease in desmosomal junctions, and widening of intercellular spaces, at both the autocrine and paracrine levels; these are associated with a reduction in E-cadherin and an increase in β-catenin [10]. These results show that SERPINB3 can trigger epithelial–mesenchymal transition, contributing to invasion and metastasis of cancer cells [10]. SERPINB3 has also been shown as a negative regulator of programmed cell death by protecting against leaked lysosomal proteases [11] and by reducing cytochrome C-dependent caspase 3 and 9 activity [83]. Yuan et al. [84] observed that upregulated SERPINB4 in IMR-90 and NHBE cells can markedly inhibit proliferative capacity and induction of the cyclin-dependent kinase inhibitor p16Ink4a and enhance the appearance of cellular senescence features including enlarged cell size and strong SA-ß-gal staining. SERPINB4 inhibition could suppress its effect on the appearance of several important cellular senescence hallmarks. Together, these results suggest that there is a necessity of SERPINB4 induction for the initiation of senescence programming.

SCCA1 and SCCA2 are upregulated by oncogenic Ras via MAPK and the ETS transcription factor PEA3 [85], which can lead to inhibition of protein turnover and an unfolded protein response and is essential for Ras-mediated NF-κB activation, cytokine production, and tumor growth [85,86]. Additionally, SERPINB3 expression has recently been found to be upregulated during hypoxia through HIF-2a-dependent mechanisms [64]. Upregulation of SCCA1 in hepatoblastoma positively correlated with Myc expression [87] and is associated with TGF-β1 and cytoplasmic β-catenin in HCC with poor prognosis [88].

Second, abnormal expression of SCCAs is reportedly related to immune cells and responses. SERPINB3 and SERPINB4 are upregulated in memory Th2 and innate helper 2 cells of allergy patients. Silencing of SERPINB3 and SERPINB4 can decrease the viability of allergenic memory Th2 cells [89] and the expression of pro-inflammatory marker, S100A8, in human keratinocytes. These reports support a role of SERPINB3 and SERPINB4 in the initiation of the acute inflammatory response [90] and provide a therapeutic approach for allergic diseases by ablation of allergic memory TH2 cells through SERPINB3 and SERPINB4. SERPINB3 can attenuate apoptosis by contrasting cytochrome c release from the mitochondria and via an antichemotactic effect in NK cells [91]. SERPINB3 and SERPINB4 mutations may exert immunogenic effect and help initiate immune response and can later be reinvigorated through checkpoint blockade [92]. The involvement of SERPINB3 in defective programmed cell death, a critical feature of autoimmunity, warrants further studies.

Moreover, SERPINB3 and SERPINB4 can drive the serpin-derived protein Pso p27, an autoantigen correlated to disease activity that contributes to inflammation in skin lesions, through non-canonical cleavage via mast cell chymase [93].

## 6. Conclusions

Previous studies have affirmed the clinical value of the tumor marker SCCA either for diagnosis or monitoring of the response to radiotherapy or chemotherapy, tumor relapse, and failure of treatment, albeit with some contrasting results because of:

- Differences in the primary site or stage distribution;
- Different measurement methods;
- Different cutoff values for SCCA;
- Different distributions of SCCA isoforms, including SCCA1, SCCA2, and SCCA–IgM;
- Ethnic biases.

Thus, the diagnostic and prognostic roles of SCCA remain controversial. To reduce the clinical heterogeneity across studies and provide more accurate and reliable comparability, a standardized detection method, scoring system, and cutoff level are warranted. Despite comparable performances between different methods, the same method should always be employed for the dynamic observation of marker kinetics.

## Data Availability

Not applicable.
